# Specific IgE to fish extracts does not predict allergy to specific species within an adult fish allergic population

**DOI:** 10.1186/2045-7022-4-27

**Published:** 2014-09-01

**Authors:** Karlijn JG Schulkes, Rob JB Klemans, Lidy Knigge, Marjolein de Bruin-Weller, Carla AFM Bruijnzeel-Koomen, Åsa Marknell deWitt, Jonas Lidholm, André C Knulst

**Affiliations:** 1 Department of Dermatology/Allergology, University Medical Center Utrecht, PO Box 85500, Utrecht 3508, GA the Netherlands; 2 Thermo Fisher Scientific, Uppsala, Sweden

**Keywords:** Fish allergy, Fish species, Food allergy, Sensitization, Specific IgE

## Abstract

**Background:**

Fish is an important cause of food allergy. Studies on fish allergy are scarce and in most cases limited to serological evaluation. Our objective was to study patterns of self-reported allergy and tolerance to different commonly consumed fish species and its correlation to IgE sensitization to the same species.

**Methods:**

Thirty-eight adult fish allergic patients completed a questionnaire regarding atopy, age of onset and symptoms to 13 commonly consumed fish species in the Netherlands (pangasius, cod, herring, eel, hake, pollock, mackerel, tilapia, salmon, sardine, tuna, plaice and swordfish). Specific IgE to these fish extracts were analyzed by ImmunoCAP.

**Results:**

Median age of onset of fish allergy was 8.5 years. Severe reactions were reported by the majority of patients (n = 20 (53%) respiratory and of these 20 patients, 6 also had cardiovascular symptoms). After diagnosis, 66% of the patients had eliminated all fish from their diet. Allergy to all species ever tried was reported by 59%. In relation to species ever tried, cod (84%) and herring (79%) were the most frequently reported culprit species while hake (57%) and swordfish (55%) were the least frequent. A positive sIgE (value ≥ 0.35 kU_A_/L) to the culprit species ranged between 50% (swordfish) and 100% (hake). In tolerant patients, a negative sIgE (value < 0.35 kU_A_/L) ranged from 0% (hake, pollock and swordfish) to 75% (sardine). For cod, the agreement between sIgE test results and reported allergy or tolerance was 82% and 25%, respectively. Sensitization to cod parvalbumin (Gad c 1) was present in 77% of all patients.

**Conclusion:**

Serological cross-reactivity between fish species is frequent, but in a significant proportion of patients, clinical relevance appears to be limited to only certain species. A well-taken history or food challenge is required for discrimination between allergy to the different fish species.

## Background

Fish is known for its potential to induce severe allergic reactions, ranging from mild local to severe respiratory or even cardiovascular symptoms [1-3]. It is one of the twelve foods for which labelling is mandatory in the European Union [4]. The current prevalence of fish allergy in the US and Europe is estimated to be 0.1-0.5% [5-7]. A multicenter study in emergency departments suggested that 10% of food allergic reactions were caused by fish [8]. A review of published data showed that the prevalence of seafood allergy is higher in regions of the world were fish is more frequently consumed, for example in Malaysia, Thailand and Singapore [6]. Reported studies on fish allergy have mostly been restricted to serological evaluation [3,9-16].

Until recently, assessment of sensitization to fish was only based on the use of whole fish muscle extract. Several allergenic components in fish have been characterized [17-22]. Parvalbumin has been described as a major fish allergen and can be found in great amount in the fast skeletal muscles [17,20]. In cod, this 12 kDa allergen was first identified in 1968 and later named Gad c 1 [17,20]. Parvalbumins have also been described in salmon (Sal s 1), mackerel (Sco a 1, Sco s 1 and Sco j 1), carp (Cyp c 1), pollock and several tropical fish species [17-22]. Previous studies have demonstrated the extensive serological cross-reactivity between cod and many other fish species, corroborating the widespread occurrence and similarity of parvalbumin among these species [3,9-16]. For this reason, fish allergic patients are often advised to avoid all fish. In addition to parvalbumin, IgE antibody binding to a 41 kD cod protein (referred to as “Gad c 41 k” in this paper), identified as aldehyde phosphate dehydrogenase (APDH) has been observed in a minority of fish allergic patients [21,22]. In addition to parvalbumin, the major fish allergen, other fish allergens have been isolated. Examples of these less well-known allergens are; the hormone vitellogenin from the Beluga caviar, collagen and gelatin isolated from the skin and muscle tissues of the fish [23-27].

Until now, it remains unclear to what extent observed serological cross-reactivity among different fish species is accompanied by clinical cross-reactivity. Evidence regarding clinical cross-reactivity between fish species is scarce and it is therefore possible that common recommendations to patients to avoid all fish may represent an unjustified and unnecessary dietary restriction. The aim of this study was to assess the relationship between IgE sensitization and self-reported allergic reactions to 13 different fish species in a fish allergic population.

## Methods

### Patients

Adult fish allergic patients (n = 43) were recruited from the outpatient clinic of the Department of Dermatology/Allergology at the University Medical Centre Utrecht. Patients were included if they reported allergic symptoms after ingestion of fish in combination with a positive challenge and/or sensitization; a sIgE level to cod ≥ 0.35 kU_A_/L and/or a positive skin prick test (SPT) to at least one of the tested fish species. Patients were excluded if they did not respond to the questionnaire.

In addition, 15 fish tolerant atopic controls were included (5 birch and grass pollen allergic, 5 grass pollen allergic and 5 allergic to foods other than fish). The study was approved by Utrecht Medical Centre Utrecht Medical Research Ethics Committee.

### Skin prick test and food challenge

SPT was performed in 13 patients (ALK-ABELLO, Nieuwegein, the Netherlands). As positive and negative controls, histamine dihydrochloride 10 mg/mL and glycerol diluent were used, respectively. A skin reaction ≥ 3 mm than negative control was considered positive. Depending on each patient’s history, SPT was performed with cod, hake, sardine, eel, sole, tuna, salmon, plaice, herring or whiting.

Open fish challenges with tuna, cod, halibut and tilapia were performed in 4 patients with doses ranging from 5 to 150 gram. The challenge was considered positive if objective symptoms occurred; rhinoconjunctivitis, angioedema, urticaria, emesis, diarrhoea, hoarseness, dyspnoea, stridor and/or tachycardia.

### Questionnaire

A standardized questionnaire was provided to all patients concerning general allergy characteristics and fish allergy in particular. Patients were asked to provide information on the species they tried, symptoms of intolerance and dietary avoidance of 13 fish species commonly consumed in the Netherlands (pangasius, cod, herring, eel, hake, pollock, mackerel, tilapia, salmon, sardine, tuna, plaice and swordfish). For our analyses, respiratory or cardiovascular symptoms were considered as severe and all other symptoms as mild. In addition, questions concerning other food allergies, atopic dermatitis, allergic rhinitis and asthma were asked.

### IgE reactivity

sIgE to the whole extract of the 13 fish species mentioned above, as well as to rGad c 1 and nGad c 41 k were analyzed by ImmunoCAP (Thermo Fisher Scientific, Uppsala, Sweden). Values ≥ 0.35 kU_A_/L were considered positive. Gad c 41 k was purified essentially as described except that the ion exchange chromatography step was performed using Q Sepharose (GE Healthcare Life Sciences, Uppsala, Sweden) instead of hydroxyapatite and size exclusion chromatography on Superdex G75 was used as a final polishing step instead of preparative electrophoresis [22]. Experimental ImmunoCAP tests were prepared as described [28].

### Statistics

Descriptive statistics were presented as numbers with percentages or as median values with interquartile ranges. Differences between groups were analyzed with the Mann–Whitney U test. All analyses were performed with SPSS (version 16.0; SPSS Inc, Chicago).

## Results

### Patient characteristics and reactivity to fish, shellfish and other foods

Forty-three adult fish allergic patients were first identified on the basis of reported symptoms after the ingestion of fish, in combination with concordant sensitization (n = 39) or a positive challenge (n = 4). Thirty-eight of 43 patients (88%) completed the questionnaire and were included in the study. Table 1 shows the clinical characteristics of all study subjects. Their median age was 33 years and 17/38 (45%) were males. Patients were highly atopic; 55% (21/38) of the patients reported a history of allergic asthma, allergic rhinitis and atopic dermatitis. The most frequently self-reported other food allergies were to peanut (55%), hazelnut (47%) and walnut (42%).

**Table 1:** Patient characteristics

	**Total (n = 38)**
Male	17 (45%)
Median age (yr)	33 (17–71)
Asthma	27 (71%)
Allergic rhinitis	32 (84%)
Atopic dermatitis	32 (84%)
**Other reported food allergies**	
Peanut	21 (55%)
Hazelnut	18 (47%)
Walnut	16 (42%)
Shrimp	12 (32%)
Crab	12 (32%)
Lobster	10 (26%)

Data are presented as numbers and percentages or median values with interquartile ranges.

### Clinical symptoms and course of fish allergy

The median age at which patients reported to have their first allergic reaction to fish was 8.5 years. All patients reported oral symptoms during their most severe reaction, skin symptoms were reported by 68% (26/38), gastro-intestinal symptoms by 55% (21/38), respiratory symptoms by 53% (20/38) and of the 20 patients with respiratory symptoms 6 reported also cardiovascular symptoms. One patient reported only oral symptoms as most severe symptom. In the majority of patients (76%, n = 29), the reaction started within 5 minutes after ingestion. After diagnosis, 25 of 38 (66%) patients eliminated all fish from their diet and 11 patients (29%) only avoided the species that had caused an allergic reaction. Two patients with a mild allergy to cod or tuna, resulting in oral symptoms, did not eliminate any fish from their diet. Of the 25 patients that eliminated all fish from their diet, 16 reported severe symptoms (cardiovascular of respiratory). In contrast, 6 out of 11 patients that eliminated only the symptom giving species reported severe symptoms.

### Fish species eliciting allergic symptoms

**Figure 1:**
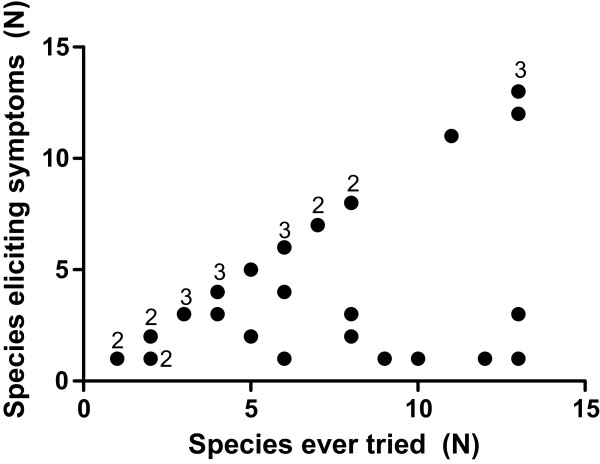
**Number of fish species eliciting symptoms vs. number of species ever consumed.** The numbers in the graph represent the data points with multiple patients.

The number of fish species causing symptoms in relation to the number of species ever consumed in the study population is shown in Figure 1. Allergy to all fish species ever tried was reported by 20 (59%) and allergy to a single species by 7 (21%). Patients of the latter subgroup reacted to cod (n = 4), tuna (n = 2) or herring (n = 1). The remaining 7 patients (21%) reported allergy to more than one species but were able to eat other species without symptoms. The patients allergic to all species ever consumed had tried on average 7 species, whereas both the mono-allergic group and the group reporting allergy to several but not all fish ever consumed had tried on average 8 species. Two patients reported allergy to one species but had never tried other species. Two patients provided incomplete information.

In relation to species ever tried, symptoms were most commonly reported to cod (84%) and herring (79%), and least commonly to tuna (58%), hake (57%) and swordfish (55%, Table 2). There was no correlation between severity of symptoms and number of fish species eliciting an allergic reaction. In addition, no correlation could be observed between the severity of symptoms and species of fish causing the reaction.

**Table 2:** Fish species eliciting allergic symptoms in relation to species ever tried (n = 38)

	Consumed*	Allergic symptoms**	Unknown if ever tried*
Cod	32 (84%)	27 (84%)	-
Salmon	30 (79%)	20 (67%)	-
Eel	22 (58%)	17 (77%)	1 (3%)
Herring	19 (50%)	15 (79%)	1 (3%)
Pollock	20 (53%)	14 (70%)	1 (3%)
Tuna	24 (63%)	14 (58%)	-
Mackerel	16 (42%)	11 (69%)	-
Plaice	18 (47%)	11 (61%)	-
Pangasius	14 (37%)	10 (71%)	1 (3%)
Tilapia	15 (39%)	10 (67%)	1 (3%)
Sardine	15 (39%)	9 (60%)	1 (3%)
Swordfish	11 (29%)	6 (55%)	1 (3%)
Hake	7 (18%)	4 (57%)	3 (8%)

Data are presented as numbers and percentages.

*Percentage of all included patients n = 38.

**Percentage of consumed.

### Sensitization profile to 13 different fish species and parvalbumin

An extensive serological evaluation was performed in 32 of the 38 patients while serum was lacking from 6 patients. No significant differences were found between these 6 patients and the other 32 patients with regard to age, severity of reaction, age of onset and number of fish species consumed.

**Figure 2:**
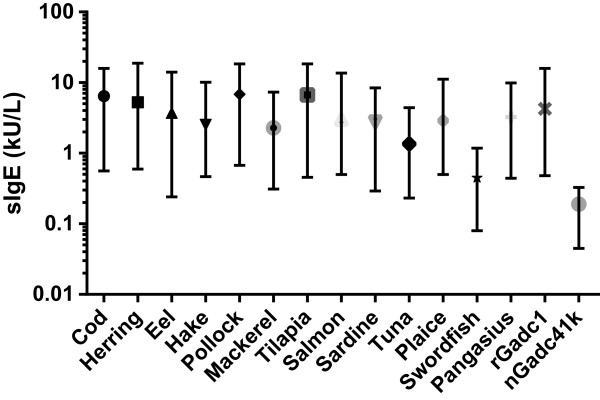
**Median values and interquartile ranges of sIgE to 13 different fish species (n = 32).** * = P < .001, ** = P < .05.

Figure 2 shows the median levels and interquartile ranges of sIgE antibodies to 13 different fish extracts and the cod allergen components Gad c 1 and Gad c 41 k of all 32 patients. In comparison to cod, with a median sIgE level of 6.4 kU_A_/L, significantly lower levels of sIgE were observed for tuna (1.0 kU_A_/L, *P* = 0.03) and swordfish (0.5 kU_A_/L, *P* = 0.003). The median level of sIgE to Gad c 1 (4.5 kU_A_/L) was comparable to that of whole cod extract whereas Gad c 41 k only showed weak IgE binding in a few patients’ sera (median 0.2 kU_A_/L).

Median sIgE levels in the allergic versus the non-allergic patient group, species by species, are listed in Table 3. Patients were considered non-allergic if they had eaten the specific fish species without symptoms. Although a trend of higher sIgE values to most fish extracts was seen in the allergic group, no significant differences were observed. In addition, no significant differences in sIgE levels to specific fish extracts were found between subjects with self-reported mild or severe allergic reactions (data not shown).

**Table 3:** Median specific IgE levels in allergic vs. non-allergic patients (n = 32*)

	**Allergic patients**	Non-allergic patients**	***P*-value**	**Se**	**Sp**	**Atopic controls (n = 15)**	
	**sIgE kU/L (IQR)**	**sIgE kU/L (IQR)**				**sIgE (IQR) kU**_**A**_**/L**	Sp***
Cod	9.2 (0.8-17.4)	0.9 (0.3-3.4)	0.16	82	25	0.03 (0.01-0.06)	93
Salmon	2.9 (0.5-21.3)	1.6 (0.4-3.1)	0.41	81	25	0.01 (0.00-0.20)	93
Eel	0.7 (0.1-22.1)	0.5 (0.1-2.7)	0.71	63	50	0.00 (0.00-0.03)	93
Herring	6.0 (0.4-43.7)	6.2 (0.3-7.5)	0.61	79	67	0.00 (0.00-0.09)	93
Pollock	6.9 (1.0-32.5)	1.5 (0.8-6.0)	0.52	69	0	0.01 (0.00-0.10)	87
Tuna	2.0 (0.1-4.7)	0.7 (0.0-2.7)	0.50	67	43	0.02 (0.01-0.07)	93
Mackerel	0.5 (0.0-14.7)	0.3 (0.0-1.6)	0.43	64	50	0.01 (0.00-0.04)	93
Plaice	0.9 (0.1-24.2)	0.4 (0.1-2.7)	0.44	67	50	0.02 (0.01-0.07)	87
Pangasius	5.0 (0.7-26.8)	0.3 (0.1-1.0)	0.12	89	67	0.00 (0.00-0.05)	93
Tilapia	0.9 (0.0-31.5)	0.4 (0.1-3.1)	0.59	67	60	0.01 (0.00-0.04)	93
Sardine	7.9 (0.1-4.7)	1.0 (0.2-2.2)	0.54	56	75	0.01 (0.00-0.04)	93
Swordfish	1.3 (0.0-64.4)	0.9 (0.4-4.6)	0.72	50	0	0.05 (0.03-0.10)	87
Hake	18.9 (0.7-92.3)	2.1 (0.4-3.7)	0.36	100	0	0.04 (0.03-0.20)	87

*IQR*, interquartile range; *Se,* Sensitivity; *Sp,* Specificity.

*Only patients of whom serum was analyzed (32 of 38 patients).

**Patients were considered non-allergic if they had eaten the specific fish species without symptoms.

***Specificity when only analyzing the atopic control group.

### Discrimination between allergy to different fish species

Cod sIgE ≥ 0.35 kU_A_/L was present in 18 of 22 patients reporting allergy to cod of whom serum could be analyzed and in 3 of 4 fish allergic patients that reported tolerance to cod, corresponding to a sensitivity of 82% and an inter-species specificity of 25% (Table 3).

When using the same cut-off value of ≥ 0.35 kU/L, the sensitivity of sIgE to individual fish extracts ranged between 50% for swordfish and 100% for hake (Table 3). It should be noted however, for both species there were only a few allergic patients. Inter-species specificity ranged between 0% for hake, pollock and swordfish and 75% for sardine. sIgE to Gad c 1 was recognized by 77% of all fish allergic patients. In regard to the fish tolerant atopic control group (n = 15), specificity values ranged from 87% for pollock to 93% for herring, mackerel, pangasius and sardine (Table 3).

SPT data were available for 13 patients included in this study. Cod was the only fish species that was tested in all those 13 patients and median wheal diameter was 7.5 mm (IQR 1.8 – 10.0 mm). There was no significant difference between patients with cod allergy (n = 8) and patients who tolerated cod (n = 3): 8.5 vs. 2.0 mm, *P* = 0.19. Two patients reported they had never eaten cod. The SPT with cod had a sensitivity of 75% (6/8) and an inter-species specificity of 33% (1/3) when using a cut-off value ≥ 3 mm. Too few observations were available for statistical analysis of SPT results for the other fish species.

## Discussion

This study described clinical characteristics in combination with sIgE to different fish extracts within a large adult fish allergic population. Other studies describing fish allergic populations mainly focused either on serological evaluation or clinical symptoms, described small populations, focused on other serological markers or were performed in children [3,9-22,29-33]. The present study focused on the pattern of self-reported allergy and tolerance to different commonly consumed fish species and its correlation to IgE sensitization to the same fish extracts.

The median age of onset of fish allergy in our population was 8.5 years. This is relatively high in comparison with a study of Connett et al., which was performed in Singapore and the Philippines [34]. This can be attributed to the higher age at which fish is typically introduced in children’s diet in Europe. In Asian diets, the first intake of seafood seems to be very early in life, with an average of 7 months [35].

More than half of the patients reported severe symptoms to fish. This is in line with a study of Sicherer et al., who performed a random telephone survey on fish allergy in the United States [5]. Dyspnoea or throat tightness was reported by 50% in that study. Connett et al. reported that in the Philippines, 16% of the children with fish allergy experienced throat tightness, 11% wheezing and 8% loss of consciousness [34]. A clear limitation of our study was that symptoms to the 13 specific fish species were based on self-reported symptoms. However, allergy to at least one fish species was diagnosed and confirmed by a physician in all patients.

In the adult fish allergic population studied here, only 59% of the patients reported a reaction to all fish species ever tried, indicating that a considerable proportion of the patients tolerated one or more species. This is consistent with data from other studies [9,10,35] and it has been suggested that differential clinical reactivity to various fish species could be related to their content of parvalbumin [17-22]. In contrast, one study described a patient with an isolated allergy to swordfish whose IgE antibodies bound to a swordfish specific allergen but not to parvalbumin [36]. This observation suggests that, apart from parvalbumin, species specific fish determinants may exist and play a role. Another possibility for the cross-reactivity is earlier emphasized by Kuehn et al. [15]. They reported that there might be other proteins as enolases, aldolases and fish gelatin, which might contribute to serological cross-reactivity.

In our study, however, we found no significant difference in occurrence or concentration of sIgE to Gad c 1 between patients with allergy to a single species, several species or all species ever tried.

Diagnostic tools for evaluating adult fish allergic patients in daily clinical practice are sIgE tests and SPTs. In this study, we found that median levels of sIgE to most fish extracts were not significantly different compared to cod, which is most often used in diagnostics. Two exceptions were tuna and swordfish, to which significantly lower sIgE levels were observed. This might be related to the lower IgE reactivity to the major allergen parvalbumin in the patients allergic to tuna and swordfish. Another possible explanation is that parvalbumin is structurally different or less abundant in tuna as compared to other fish species [3,12-16].

Our results demonstrated that sIgE to different fish extracts did not correlate well to reported allergy or tolerance to those species among the patients of the fish allergic population studied. Thus, it appears that serological cross-reactivity between fish species is not necessarily associated with clinical cross-reactivity. Available data for SPT were incomplete but indicated that also skin test results did not provide for discrimination in regard to fish species. This would suggest that assessment of sensitization to different fish species in patients already diagnosed with allergy to one fish species does not provide enough information regarding allergy or tolerance to these specific species. Therefore, a well-taken history is essential. We would like to emphasize that the role of sIgE in diagnosing fish allergy in general was not studied. Such a study would need another design. However, the low frequency of detectable IgE in the fish tolerant control group demonstrated high specificity of sIgE testing when used as a diagnostic tool for fish allergy in general.

The strength of this study was that the fish allergy of all patients was thoroughly evaluated by a standardized questionnaire focusing on 13 species that are most commonly consumed in our country. In addition, their clinical history could be compared to the sensitization pattern. Although symptoms to the 13 specific fish species were based on self-reported symptoms, allergy to at least one fish species was diagnosed and confirmed by a physician in all patients. A food challenge with other fish species however, could possibly have provided more accurate information. Another possible limitation of the study was the fact that SPT data were incomplete. Therefore, the value of the SPT could not be accurately assessed.

In conclusion, fish allergy in the adult population studied here was mostly severe in nature. Serological cross-reactivity occurred in most patients, but 41% tolerated at least one fish species. A well-taken history is essential in a subject that has been diagnosed with fish allergy since sIgE reactivity did not predict the pattern of allergy to different fish species.

## Abbreviations

kD: kilo Dalton; sIgE: Specific immunoglobulin E; SPT: Skin prick test.

## Competing interest

A Marknell DeWitt and J Lidholm are employed by Thermo Fisher Scientific (Uppsala, Sweden), manufacturer of the IgE assay system used in this study. The other authors reported no possible competing interest.

## Authors’ contributions

KJGS: Substantial contributions to conception and design, acquisition, analysis and interpretation of data, drafting article and final approval of version to be published. RJBK: Substantial contributions to conception and design, acquisition, analysis and interpretation of data, drafting article and final approval of version to be published. LK: Substantial contributions to conception and design, acquisition, analysis and interpretation of data, drafting article and final approval of version to be published. MdeB-W: Substantial contributions to conception and design, revising the article critically for important intellectual content and final approval of version to be published. CAFMB-K: Substantial contributions to conception and design, revising the article critically for important intellectual content and final approval of version to be published. ÅMDeW: Substantial contribution to acquisition of data, revising the article critically for important intellectual content and final approval of version to be published. JL: Substantial contributions to conception and design, acquisition and interpretation of data, revising the article critically for important intellectual content and final approval of version to be published. ACK: Substantial contributions to conception and design, interpretation of data, revising the article critically for important intellectual content and final approval of version to be published.

## References

[B1] Yunginger JW Sweeney KG Sturner WQ Giannandra LA Teigland JD Bray M Benson PA York JA Biedrzycki L Squillace DL Helm RM Fatal food-induced anaphylaxis JAMA 1988 260 1450 1452 10.1001/jama.1988.03410100140041 3404604

[B2] Bock SA Muñoz-Furlong A Sampson HA Fatalities due to anaphylactic reactions to foods J Allergy Clin Immunol 2001 107 191 193 10.1067/mai.2001.112031 11150011

[B3] Helbling A Haydel R McCants ML Musmand JJ El Dahr J Lehrer SB Fish allergy: is cross-reactivity among fish species relevant? A double-blind placebo-controlled food challenge studies of fish allergic adults Ann Allergy Asthma Immunol 1999 83 517 523 10.1016/S1081-1206(10)62862-1 10619342

[B4] Humières J Wal JM EU regulation: what’s new in terms of labelling of food allergens? Allergy 2004 59 12 1259 1261 10.1111/j.1398-9995.2004.00618.x 15507092

[B5] Sicherer SH Munoz-Furlong A Sampson HA Prevalence of seafood allergy in the United States determined by a random telephone survey J Allergy Clin Immunol 2004 114 1 159 165 10.1016/j.jaci.2004.04.018 15241360

[B6] Rona RJ Keil T Summers C Gislason D Zuidmeer L Sodergren E Sigurdardottir ST Lindner T Goldhahn K Dahlstrom K Mc Bride D Madsen C The prevalence of food allergy: a meta-analysis J Allergy Clin Immunol 2007 120 3 638 646 10.1016/j.jaci.2007.05.026 17628647

[B7] 1961–2007 Fish and fishery products world apparent consumption based on food balance sheets [Internet] 2011 Rome, Italy: Food and Agriculture Organization of the Unitated Nations [cited 2012 June 8] Available from: ftp://ftp.fao.org/FI/CDrom/CD_yearbook_2009/root/food_balance/introduction.pdf

[B8] Clark S Bock SA Gaeta TJ Brenner BE Cydulka RK Camargo CA Multicenter Airway Research Collaboration-8 Investigators. Multicenter study of emergency department visits for food allergies J Allergy Clin Immunol 2004 113 2 347 352 10.1016/j.jaci.2003.10.053 14767453

[B9] Hansen TK Bindslev-Jensen C Skov PS Poulsen LK Codfish allergy in adults: IgE cross-reactivity among fish species Ann Allergy Asthma Immunol 1997 78 2 187 194 10.1016/S1081-1206(10)63386-8 9048527

[B10] Hansen TK Bindslev-Jensen C Skov PS Poulsen LK Codfish allergy in adults. Specific tests for IgE and histamine release vs double-blind, placebo-controlled challenges Clin Exp Allergy 1996 26 11 1276 1285 10.1111/j.1365-2222.1996.tb00525.x 8955577

[B11] Hansen TK Bindslev-Jensen C Codfish allergy in adults. Identification and diagnosis Allergy 1992 47 6 610 617 10.1111/j.1398-9995.1992.tb02383.x 1283656

[B12] Helbling A McCants ML Musmand JJ Schwartz HJ Lehrer SB Immunopathogenesis of fish allergy: identification of fish-allergic adults by skin test and radioallergosorbent test Ann Allergy Asthma Immunol 1996 77 1 48 54 10.1016/S1081-1206(10)63479-5 8705636

[B13] Sten E Hansen TK Stahl Skov P Andersen SB Torp A Bindslev-Jensen U Bindslev-Jensen C Poulsen LK Cross-reactivity to eel, eelpout and ocean pout in codfish-allergic patients Allergy 2004 59 11 1173 1180 10.1111/j.1398-9995.2004.00497.x 15461598

[B14] Pascual C Martín Esteban M Crespo JF Fish allergy: evaluation of the importance of cross-reactivity J Pediatr 1992 121 S29 S34 10.1016/S0022-3476(05)81403-9 1447631

[B15] Kuehn A Hilger C Lehners-Weber C Codreanu-Morel F Morisset M Metz-Favre C Pauli G de Blay F Revets D Muller CP Vogel L Vieths S Hentges F Identification of enolases and aldolases as important fish allergens in cod, salmon and tuna: component resolved diagnosis using parvalbumin and the new allergens Clin Exp Allergy 2013 43 7 811 822 10.1111/cea.12117 23786287

[B16] Griesmeier U Va’zquez-Corte’s S Bublin M Radauer C Ma Y Briza P Ferna’ndez-Rivas M Breiteneder H Expression levels of parvalbumins determine allergenicity of fish species Allergy 2010 65 191 198 10.1111/j.1398-9995.2009.02162.x 19796207

[B17] Elsayed S Bennich H The primary structure of allergen M from cod Scand J Immunol 1975 4 2 203 208 10.1111/j.1365-3083.1975.tb02618.x 1145128

[B18] Bernhisel-Broadbent J Scanlon SM Sampson HA Fish hypersensitivity. I. In vitro and oral challenge results in fish-allergic patients J Allergy Clin Immunol 1992 89 3 730 737 10.1016/0091-6749(92)90381-B 1545094

[B19] Van Do T Hordvik I Endresen C Elsayed S Characterization of parvalbumin, the major allergen in Alaska pollack, and comparison with codfish Allergen M Mol Immunol 2005 42 3 345 353 10.1016/j.molimm.2004.09.001 15589323

[B20] Lim DL Neo KH Yi FC Chua KY Goh DL Shek LP Giam YC van Beever HP Lee BW Parvalbumin - the major tropical fish allergen Pediatr Allergy Immunol 2008 19 5 399 407 10.1111/j.1399-3038.2007.00674.x 18221468

[B21] Das Dores S Chopin C Romano A Galland-Irmouli AV Quaratino D Pascual C Fluerence J Guéant JL IgE-binding and cross-reactivity of a new 41 kDa allergen of codfish Allergy 2002 57 84 87 10.1034/j.1398-9995.57.s72.6.x 12144562

[B22] Galland AV Dory D Pons L Chopin C Rabesona H Gueant JL Fluerence J Purification of a 41 kDa cod-allergenic protein J Chromatogr B Biomed Sci Appl 1998 706 63 71 10.1016/S0378-4347(97)00457-X 9544808

[B23] Escudero R Gamboa PM Anton J Sanz ML Food allergy due to trout roe J Invest Allerg and Clin 2007 17 346 347 17982930

[B24] Perez-Gordo M Sanchez-Garcia S Cases B Pastor C Vivanco F Cuesta-Herranz J Identification of vitellogenin as an allergen in Beluga caviar allergy Allergy 2008 63 479 480 10.1111/j.1398-9995.2007.01614.x 18315737

[B25] Hamada Y Nagashima Y Shiomi K Identification of collagen as a new fish allergen Biosci Biotech Bioch 2001 65 285 291 10.1271/bbb.65.285 11302160

[B26] Sakaguchi M Toda M Ebihara T Irie S Hori H Imai A Yanagida M Miyazawa H Ohsuna H Ikezawa Z Inouye S IgE antibody to fish gelatin (type I collagen) in patients with fish allergy J Allergy Clin Immunol 2000 106 579 584 10.1067/mai.2000.108499 10984381

[B27] Taylor SL Kabourek JL Hefle SL Fish allergy: fish and products thereof J Food Sci 2004 69 R175 R180 10.1111/j.1750-3841.2004.tb18022.x

[B28] Marknell DA Niederberger V Lehtonen P Spitzauer S Sperr WR Valent P Valenta R Lidholm J Molecular and immunological characterization of a novel timothy grass (Phleum pratense) pollen allergen, Phl p 11 Clin Exp Allergy 2002 32 1329 1340 10.1046/j.1365-2222.2002.01467.x 12220472

[B29] de Martino M Peruzzi M de Luca M Amato AG Galli L Lega L Azzari C Vierucci A Fish allergy in children Ann Allergy 1993 71 2 159 165 8346870

[B30] Ng IE Turner PJ Kemp AS Campbell DE Parental perceptions and dietary adherence in children with seafood allergy Pediatr Allergy Immunol 2011 22 720 728 10.1111/j.1399-3038.2011.01189.x 21749460

[B31] Tsabouri S Triga M Makris M Kalogeromitros D Church MK Priftis KN Fish and shellfish allergy in children: Review of a persistent food allergy Pediatr Allergy Immunol 2012 23 7 608 615 10.1111/j.1399-3038.2012.01275.x 22554093

[B32] Pascual CR Reche M Fiandor A Valbuena T Cuevas T Esteban MM Fish allergy in childhood Pediatr Allergy Immunol 2008 19 573 579 10.1111/j.1399-3038.2008.00822.x 18950323

[B33] Turner P Ng I Kemp A Campell D Seafood allergy in children: a descriptive study Ann Allergy Asthma Immunol 2011 106 494 501 10.1016/j.anai.2011.02.001 21624749

[B34] Connett GJ Gerez I Cabrera-Morales EA Yuenyongviwat A Ngamphaiboon J Chatchatee P Sangsupawanich P Soh SH Yap GC Sheck LP Lee BW A Population-Based Study of Fish Allergy in the Philippines, Singapore and Thailand Int Arch Allergy Immunol 2012 159 384 390 10.1159/000338940 22846665

[B35] Chiang WC Kidon MI Liew WK Goh A Tang JP Chay OM The changing face of food hypersensitivity in an Asian community Clin Exp Allergy 2007 37 1055 1061 10.1111/j.1365-2222.2007.02752.x 17581199

[B36] Kelso JM Jones RT Yunginger Monospecific allergy to swordfish Ann Allergy Asthma Immunol 1996 77 3 227 228 10.1016/S1081-1206(10)63260-7 8814049

